# The Folate Cycle As a Cause of Natural Killer Cell Dysfunction and Viral Etiology in Type 1 Diabetes

**DOI:** 10.3389/fendo.2017.00315

**Published:** 2017-11-23

**Authors:** Allison L. Bayer, Christopher A. Fraker

**Affiliations:** ^1^Immunobiology Laboratory, Leonard M. Miller School of Medicine, Diabetes Research Institute, University of Miami, Miami, FL, United States; ^2^Tissue and Biomedical Engineering Laboratory, Leonard M. Miller School of Medicine, Diabetes Research Institute, University of Miami, Miami, FL, United States

**Keywords:** diabetes, natural killer cells, virus, folic acid, folate cycle

## Abstract

The folate pathway is critical to proper cellular function and metabolism. It is responsible for multiple functions, including energy (ATP) production, methylation reactions for DNA and protein synthesis and the production of immunomodulatory molecules, inosine and adenosine. These play an important role in immune signaling and cytotoxicity. Herein, we hypothesize that defects in the folate pathway in genetically susceptible individuals could lead to immune dysfunction, permissive environments for chronic cyclical latent/lytic viral infection, and, ultimately, the development of unchecked autoimmune responses to infected tissue, in this case islet beta cells. In the context of type 1 diabetes (T1D), there has been a recent increase in newly diagnosed cases of T1D in the past 20 years that has exceeded previous epidemiological predictions with yet unidentified factor(s). This speaks to a potential environmental trigger that adversely affects immune responses. Most research into the immune dysfunction of T1D has focused on downstream adaptive responses of T and B cells neglecting the role of the upstream innate players such as natural killer (NK) cells. Constantly, surveilling the blood and tissues for pathogens, NK cells remove threats through direct cytotoxic responses and recruitment of adaptive responses using cytokines, such as IL-1β and IFN-γ. One long-standing hypothesis suggests viral infection as a potential trigger for the autoimmune response in T1D. Recent data suggest multiple viruses as potential causal agents. Intertwined with this is an observed reduced NK cell enumeration, cytotoxicity, and cytokine signaling in T1D patients. Many of the viruses implicated in T1D are chronic latent/lysogenic infections with demonstrated capacity to reduce NK cell response and number through mechanisms that resemble those of pregnancy tolerance. Defects in the folate pathway in T1D patients could result in decreased immune response to viral infection or viral reactivation. Dampened NK responses to infections result in improper signaling, improper antigen presentation, and amplified CD8^+^ lymphocyte proliferation and cytotoxicity, a hallmark of beta cell infiltrates in patients with T1D onset. This would suggest a critical role for NK cells in T1D development linked to viral infection and the importance of the folate pathway in maintaining proper NK response.

## The Cellular Folate Pathway: Role in Energy Production, Protein/DNA Synthesis, and Immune Function

**Figure 1:**
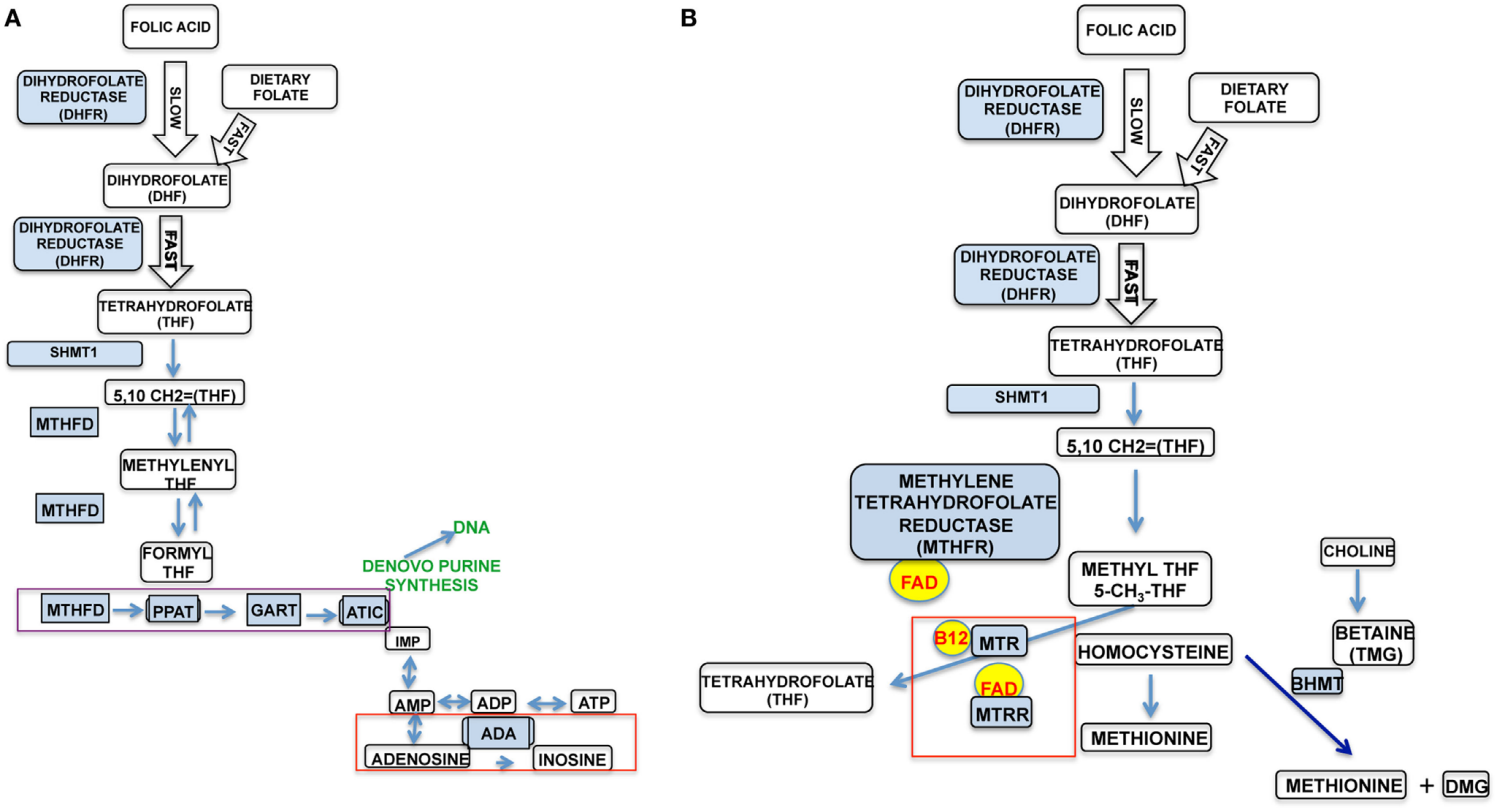
The two major pathways in the cellular folate cycle. **(A)** It details the purinosome, DNA synthesis, cellular energy pathway (ATP synthesis), and immune system modulation. **(B)** It details amino acid synthesis (homocysteine–methionine).

Figure 1 depicts the cellular folate pathway and the importance of the vitamin (B9) for the maintenance of cellular energy, DNA manufacture and repair, protein production, single-carbon transfers (methylation) and as a co-factor for numerous reactions. It is especially important for rapid cell growth and division, and critical to proper immune function. Particularly related to natural killer (NK) cell function, the production of inosine (Figure 1A, red box) is critical in maintaining NK cell cytotoxicity and proliferation in response to pathogens, while the production of adenosine will result in decreased NK cytotoxicity and proliferation, as well as generalized immunosuppression, as evidenced by adenosine deaminase (ADA) inhibitors, such as EHNA and drugs, such as Methotrexate. Literature demonstrates that increased activity of the enzymes associated with energy production (ATIC and GART), shown in Figure 1A (purple box) which (1) suppresses the function of ADA and the formation of inosine and hypoxanthine and (2) causes the internalization of the insulin receptor and an excess of intracellular ATP/adenosine (1).

In Figure 1B, important protein synthesis and methylation occurs, particularly the regeneration of the disease-associated homocysteine to methionine. Methionine is an essential amino acid critical to the formation of many biologically active proteins and the methylation of other critical products, such as S-adenosyl methionine, an important methyl donor to further methylation reactions. Defects in the folate pathway have been linked to numerous disease conditions, including fetal/infant neural tube defects, homocysteinemia, anemia, cognitive defects, cardiovascular disease, and cancer. It is clear that changes in the folate pathway could significantly impact functions throughout the whole body, including cell energy, protein/DNA synthesis, and, critically, immune function.

**Figure 2:**
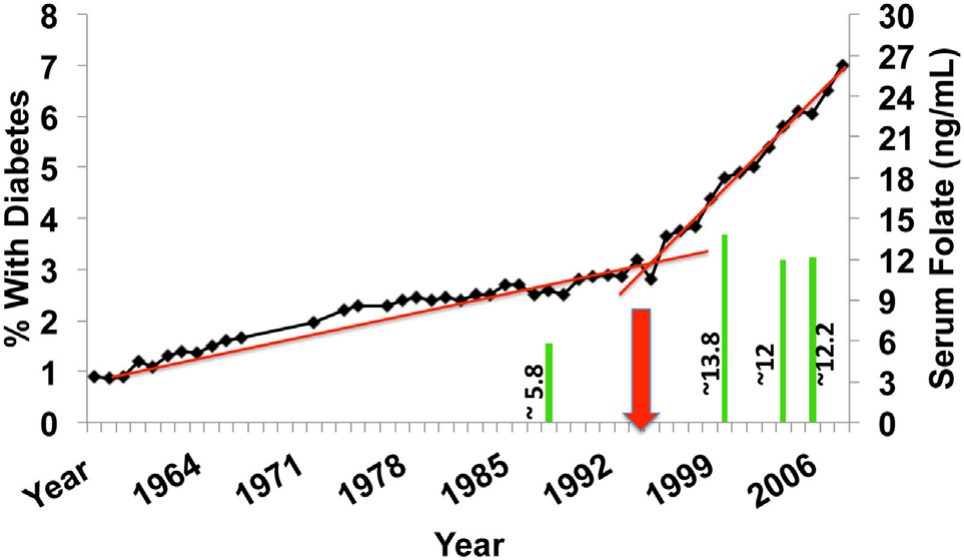
U.S. incidence of diabetes over the past 50 years (type 1 diabetes and T2D) expressed as % of total population. The red arrow depicts the initiation point of mandatory fortification of flour products with folic acid. The green bars are measurements of serum folate levels from NHANES subjects of the corresponding years.

In 1992, the WHO recognized that there are over 2 billion people worldwide that suffer from micronutrient deficiencies, such as folate. In order to combat the increasing incidence of health conditions related to these deficiencies, 159 countries implemented a micronutrient/folic acid fortification plan in primarily, processed flour products. The flour fortification initiative became mandatory in these 159 countries in 1996 (Figure 2) and was fully implemented by 1998. Intriguingly, this is the same time period that the incidence of diabetes, both Type 1 and Type 2, began an upward trend that significantly exceeded epidemiological predictions (replotted from http://cdc.gov/diabetes/statistics). Superimposed on the graphic of Figure 2 are results from the NHANES study examining serum folate levels in subjects over the age of 9 at specific time points within that same period (2). In the period from 1988 to 2000, there was a nearly 2.4-fold increase in the median serum folate of all subjects. Another study examining folate and unmetabolized folic acid (UMFA) in 2007–2008 NHANES collected serum samples found UMFA in all subjects with 33.2% of the subjects having levels greater than 1 nmol/L (3). This increase in folate/folic acid levels supports the idea that micronutrient fortification may be unnecessary in developed countries and indicates a marked consumption of enriched flour products in the U.S. As well, the increase is superimposable on the rate of diabetes increase. Adding to this argument, in developing countries, comparable increases have been observed in autoimmune conditions in the past 10–15 years (4, 5). Given the importance of the folate cycle a broad, population-wide exposure to micronutrient fortification could result in sudden, dramatic increases in unexpected pathologies, such as type 1 diabetes (T1D). There is evidence that in developed countries, where micronutrient deficiencies are much less evident, people may be consuming an excess of folic acid. This excess consumption, in genetically susceptible individuals, might result in adverse health and dysfunction of the innate immune system. As an example, a recent paper demonstrated that excess B vitamin intake, the family containing folate (B9), was correlated with increased obesity and diabetes in the studied populations (6).

Folic acid is a synthetic that works in the same pathways as naturally occurring folates because it is a substrate for the enzyme dihydrofolate reductase (DHFR), segment 1 of Figure 1. It is first processed into dihydrofolate (DHF). This reaction is up to 1,300 times slower than the metabolism of non-synthetic folates in the liver of human subjects with an inherent fivefold variation in activity among subjects (7). This is the same pathway in which the immunosuppressant Methotrexate works, through competitive inhibition of DHFR. The inefficient reaction of folic acid and DHFR could potentially mimic this immunosuppressive effect. The inhibition of DHFR by Methotrexate results in dysfunction in the purinosome (Figure 1A, purple box) and the accumulation of adenosine (Figure 1A, red box), which is immunosuppressive. This is likely due to a slow production of THF, which is the primary substrate for the cell energy/immune modulatory side of the folate cycle. Thus, high levels of folic acid and variations in DHFR activity could result in high levels of UMFA that could adversely modulate NK cell and, furthermore, other immune cell activity. In support of this, malarial infection in mice fed a high folic acid (HFA) diet was associated with decreased NK cell activity, NK cell numbers, and survival; this was not observed in mice fed a control diet (8).

## NK Cells: The First-Line Defense Against Pathogens

Natural killer cells respond to and directly kill pathogenic invaders. Their name derives from their capacity to cause cytotoxicity in cells that do not properly present the major histocompatibility complex class I (MHC-I), bound with cytoplasmic peptides, to the surveillance of the immune system; either lacking expression, expressing non-self peptides, or hyper-expressing peptides. NK cells lyse target cells directly unlike adaptive cells needing effector differentiation. Mature NK cells reside in the body prepared to respond to invaders. Cytokines, such as INFγ, granzyme, and perforin, are stored in preformed granules and rapidly released upon NK cell activation. This is different from cells of the adaptive system requiring post-activation gene transcription to achieve effector status.

In addition to direct killing, NK cells are involved in the strength and finely tuned control of adaptive immune responses. In recent studies, it has been shown that NK cells control both effector and suppressive activities of downstream responses, including those of activate or kill antigen-presenting cells and regulatory T cells, cytotoxic T lymphocytes (CTLs) T-helper (Th) cells and B-cells (9–13). This is done through direct killing or by signaling through cytokines, such as TNF-a, IFN-y, and others. The role of NK cells is the modulation of cytotoxic CD8^+^ T lymphocyte response is to control aberrant/chronic inflammatory responses avoiding unchecked cell/tissue destruction. In the context of autoimmunity and T1D, defects in NK cell function and number could play a bigger role in the observed CD8^+^ CTL infiltration of beta cells and the chronic destruction of self-tissue than originally thought. In a 2012 study by Ehlers et al., autoimmune diabetes was ameliorated by NK-cell-mediated destruction of CD8^+^ CTLs in the NOD model (13). In our preliminary work, there are stark differences in NK cell populations in NOD mice compared to age/sex-matched control strains, such as C57Bl/6 and NOR mice; particularly, at the time prior to disease onset. In the Ehlers study, incubation of conventional NK cells with IL-18 resulted in an increase in a CD117 positive subset that had a direct lytic activity against the CTLs in PD-1/PD-L1-dependent manner suggesting the importance of NKs and, likely, specific subsets of NKs in adaptive immune responses in disease development. In a 2010 study by Olson et al., it was demonstrated that NK cells, reduced GVHD in an animal model by inhibiting alloreactive response by inducing apoptosis and reducing IFN gamma production by cytotoxic T-cells (14). Although NK subpopulations were not studied, we would propose that the NK cells responsible for this intricate control of the T-cell response are likely the mouse equivalent of the CD56 bright, CD117^+^ population observed in humans.

Natural killer cells constantly circulate through the blood monitoring the classical MHC-I, or human leukocyte antigen A, B, and C (HLA-A, HLA-B, and HLA-C). If classical HLA self-antigens are properly presented, the effectors will also encounter HLA-E resulting in the inhibition of the cytotoxic activity. The non-classical human leukocyte antigen HLA-E has a specialized role in cell recognition by NK cells. HLA-E is expressed on the cell surface after binding a restricted subset of peptides, primarily those derived from leading sequence signal peptides of HLA-A, -B, and -C, and, most importantly, the non-classical Class I, HLA-G. NK cells recognize the HLA-E peptide complex and produce an inhibitory effect on the cytotoxic activity of the effectors to prevent cell lysis.

Over the past 20 years, research into NK cells has greatly expanded and analyses that typically was limited to bulk NK cells identified as CD3^−^, CD56^+^/CD16^−^ or CD56^dim^/CD16^+^, has expanded to include other important clusters of differentiation for subpopulation analysis, such as CD11b, CD27, CD57, CD7, CD69, and others. Importantly, subpopulations have been classified in terms of strong cytotoxic capabilities (CD56^dim^/CD16^+^, CD11b^+^, CD27^−^, and CD57^+^) to the highly suppressive decidual phenotype (CD56^+^/CD16^−^, CD27^−^, and CD11b^−^) (15–19). This has expanded the understanding and research into the role of NK cells in numerous autoimmune pathologies, including T1D.

## Viruses Modulate the Innate Immune System through Pregnancy Tolerance Mechanisms

One of the primary roles of NK cells is combatting viral infection through direct killing of infected cells and recruitment of adaptive responses, including memory responses, to prevent reinfection with re-exposure. Many viral pathogens have developed the ability to disrupt Class I and Class II presentation in order to avoid recognition of their antigens by the immune system (20–22). This is likely the reason why higher species evolved the adaptive immune system, as the innate system was inadequate to defend against the varied number of pathogens encountered and their capability to mutate. It is also likely that some viruses survived by exploiting pathways that impart tolerance in the placenta during pregnancy. When the innate system is functioning properly, there is a balance of effector and suppressive subpopulations of NK cells that recognize invaders through unique receptor mechanisms with both activating and inhibitory pathways. The inhibitory pathways recognize MHC-I antigen expression and preferentially shut down the effector subpopulation. If the Class 1 molecules are not present or if non-self-antigens are presented, the activating pathway is initiated and the unrecognized entity is destroyed.

During pregnancy, the mother’s innate immune response, particularly that of NK cells is dampened through placental hyperexpression of membrane-bound HLA-G and elevated plasma levels of the circulating soluble isoform (23–26). The soluble form recruits decidual NK cells to the decidua forming a immunoprotective layer around the fetus, while the membrane-bound isoform disables circulating NK effector cells by inducing apoptotic signaling and reducing cytotoxicity (24, 27). In addition, HLA-G serves to stabilize the membrane presentation of HLA–E to NK effectors another potent inhibitor of cytotoxicity. This induces cytokine and chemokine secretion conducive to tolerance induction in the placenta. HLA-G is constitutively expressed in several tissues within the adult body. Initially described in trophoblast cells of the placenta, it has subsequently been found in thymic epithelial cells, erythroblasts, corneal cells, mesenchymal stem cells, and most intriguingly, pancreatic islet beta cells (28–33). Through the flexibility of the effector and the suppressive subpopulations of NK cells, the innate immune system provides mechanisms for threat removal and self-protection, much like the two arms of the downstream adaptive immune responses. It is clear why such mechanisms, if assumed by pathogens, could be utilized to escape detection and allow for unchecked persistence in a host. This long-term escape of a virus from the innate immune response could lead to ineffective viral clearance and presence in tissues normally uninfected by pathogens. This, in turn, could result in an aggressive and unchecked adaptive immune response resulting in the destruction of self-tissue, characteristic of all autoimmune conditions. The constitutive expression of pregnancy/immune modulatory factors, such as HLA-G on some somatic cells would provide an immune-privileged site for viral evasion, even from the moment of fetal development. This hypothesis would also help to explain the disparity in the female-to-male ratio of autoimmune pathologies, as every month, when a woman menstruates, she is temporarily immunosuppressed in preparation for implantation. This has been demonstrated in studies of NK cells during both pregnancy and the menstrual cycle (34–36). The one unique exception to this rule in autoimmune pathologies is T1D, where the age of onset is earlier than other autoimmunities, frequently earlier than puberty, and the female-to-male ratio is approximately 1:1. Given the constitutive expression of HLA-G on the surface of beta cells, this is easily explained as beta cells could be an immune-privileged site for viral infection (31).

Some viruses (and other somatic invaders, such as specific cancers) implement pregnancy mechanisms to avoid detection by the innate immune system. Particular whole families of viruses have the ability to lie dormant for years, integrated into the host genome, in a latent (“lysogenic”) phase of their life cycle. This behavior is characteristic of members of the Herpes and Coxsackie virus families. Members of these viral families utilize, much like cancer cells and trophoblast cells, the host’s own signaling pathways to disable the innate immune system. Particularly, HLA-E and HLA-G are modulated by viruses, such as Epstein–Barr virus (EBV), cytomegalovirus (CMV), parvovirus-B19 (Parvo B19), herpes simplex virus type 1, and RABV26 (37–45). Many of these viruses force surface expression of HLA-E, typically occurring only with self-peptide recognition, strongly inhibiting the innate effector population (46–50). These findings have broad implications in clearance of viruses from host tissues and hint at a potential etiology for the development of many disease conditions. Viruses have been suggested as a causative agent in many autoimmune pathologies, including MS, T1D, Sjogren’s syndrome, rheumatoid arthritis, Crohn’s disease, and systemic lupus erythematosus (51–64).

Important to the theory of viral etiology and recent increases in prevalence of autoimmune pathologies, including T1D, is the passage of viruses through gametes. Originally thought to only occur with endogenous retroviral infections, there is growing evidence that other viruses can be passed in gametes by means of episomal latency (65, 66). In episomal latency, viral genes are stabilized as both linear and lariat structures floating in the cytoplasm or the nucleus, without integrating into the genome. While this makes them more susceptible to viral defenses and cellular enzymes, there is the possibility that avoidance of entering the nucleus and integration with nuclear domain 10 thereby avoiding activation of interferon is beneficial to their survival and propagation. Coupled with our proposed environmental weakening of innate immune defenses, persistent viral infections cycling through latent and lytic phases and ineffectively cleared could progress to an aberrant immune response and development of autoimmune pathologies, including T1D.

## Viruses, NK Cells, and T1D

A role for viruses as a cause for T1D has been controversial and hotly debated for decades but recent findings, the result of improved detection strategies and strong collaborative efforts, are increasing the likelihood that viruses have a greater role in the disease etiology than initially thought. Specific viruses, including but not limited to the Coxsackie family (B4, B6, and B1), the Herpes family (HHV-6, EBV, and CMV), and others (e.g., Parvo B19) have all been implicated in autoimmune disease development (45, 52, 55, 58, 60, 63, 64, 67, 68). Early discordant results were due to methodological issues (sample size, sampling frequency, assay sensitivity, and biology of viral infections) and have now been resolved through research networks and standardized protocols. Recent studies have linked genetic factors that influence T1D risk with viral infection (55, 60, 69–71). Others have demonstrated enteroviral infection can occur in beta cells resulting in cell death, in the case of acute lytic infections, and dysfunction, with more chronic infection. Acute and chronic viral responses in predisposed individuals could trigger chronic islet autoimmunity. Histological pancreatic specimens from a UK cohort of new-onset T1D patients were examined and found to have viral antigens and markers of inflammation in islets containing insulin-positive cells. This was found at a significantly higher frequency in this cohort compared to non-diabetic subjects of similar age and sex. Other clinical study data associated T1D with antibody responses to certain viral strains. One group recently utilized a high throughput immuno-proteomics methodology as a screening tool examining responses to seven viruses associated with T1D most frequently in the historical literature (72). Antibody responses to 646 viral antigens associated with the seven viruses were assessed in 42 long-standing patients relative to 42 sex and age-matched controls. Antibody response to EBV, a member of the Herpes family, was found to be significantly higher in case versus control subjects in both sex and age groups. There was also a trend toward earlier EBV infection in the case subjects. This platform is an example of improved detection methodologies that are helping to unravel the association of viruses with T1D and other autoimmune conditions. EBV is a virus that has been demonstrated in the literature to disable and suppress NK cells efficiently. This supports the idea that viruses could suppress innate response leading to unmodulated CD8^+^ T-cell responses.

Improper viral clearance and manipulation of innate response had been suggested in the development of T1D in other papers (68). This group examined donor pancreata from 6 T1D patients and 26 controls, performing histopathological analysis of the tissues looking for viral infection and lymphocyte infiltration. In 3 of the 6 T1D patients, Coxsackie B4 infection was detected through positive staining of viral capsid protein (VP1) and then DNA extraction and sequencing of infected regions. In the same patients, the islet infiltrates comprised primarily NK cells. The islets cells in this group were intact and had positive staining for insulin. In the other 3 T1D patients, no virus was observed and infiltrates were NK free and represented mainly by CD8^+^ T cells. In this group, the islets were undergoing degranulation and destruction/apoptosis. It is unlikely that at the time of death, all three patients had ongoing Coxsackie B4 infection or that the cause of death was fulminant Coxsackie infection. Rather, these data offer additional compelling evidence for inefficient clearance of an enterovirus highlighted by subclinical/latent infection and the presence of nearby NK cells. Because subpopulation analysis was not performed, it is unclear whether those NK cells had cytotoxic function or were regulatory. Viruses, much like tumors, can recruit regulatory NK cells that are much like T-regulatory cells and are immunosuppressive in their function. The observed NKs could quite possibly be from this unique population; they could also be dysfunctional NK effectors. This speaks to the study by Ehlers et al., where CD117^+^/CD56^bright^ NK cells, a known regulatory phenotype, destroyed CD8^+^ CTLs associated with diabetes onset in NOD mice. As further evidence for viral manipulation of innate immune function in T1D, a 2009 paper by Tanaka et al. (73) described MHC Class 1 hyperexpression in islet cells also positive for VP1 associated with enterovirus infection and coexpression of CXCL10 and IFN gamma. This demonstrates a persistent battle between viral suppressive mechanisms and cellular chemokine/cytokine secretion recruiting immune response to the site of viral infection (73).

Despite these findings, the role of NK in T1D is not completely understood. This is likely because the majority of prior literature has examined bulk NKs ignoring multiple subsets with important and differing immunological functions (15, 18, 74). It is well established that NK dysfunction plays a role in the pathogensis of T1D. As an example, T1D patients and NOD mice have defective NKG2D signaling which is important in activation during viral response. This is present irrespective of disease duration. In addition, NK cells in T1D patients have been shown to have defective responses to IL-2 and IL-15, lipopolysaccharide, and reduced cytotoxicity and improper, often elevated, IFNγ secretion (75). At disease onset, it has been shown that the effector population, CD56^dim^CD16^pos^, is reduced (76–78), again suggesting manipulation by viral entities or an environmental factor adversely affecting the NK effector subpopulation.

In the NOD mouse, similar trends have been observed. In one study, NK infiltration into the pancreas of NOD mice was observed before T-cell auto-reactive infiltrates (74). These NKs displayed a more immature phenotype and reduced proliferative capacity, suggesting a dysfunction and turn over similarly observed by our group in long-standing and at-risk clinical patients. These could be equivalent to the suppressive subset observed in humans that inhibit DCs and CD8^+^ CTLs. One subset of these cells produced IFN-γ spontaneously, suggesting an ongoing response, perhaps to a pathogen, such as a virus. This suggests the presence of some pathogen and an NK dysfunction/pathogen clearance problem that ultimately results in an amplified T-cell response due to aberrant IFN-γ, inability of regulatory NK cells to balance CD8^+^ CTL response and eventual autoimmunity leading to β-cell destruction.

**Figure 3:**
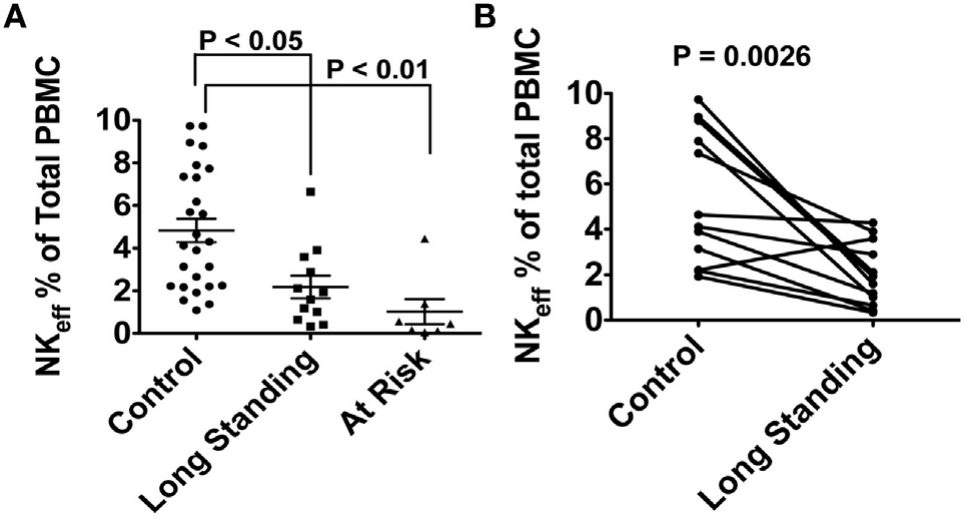
**(A)** Differences in natural killer (NK) effector cell population expressed as % of total lymphocytes in 26 control subjects, 12 long-standing type 1 diabetes (T1D) patients and 7 multiple autoantibody positive at-risk subjects. **(B)** Randomized age/sex-matching sub-analysis between control subjects and long-standing T1D patients. No correlation was found between NK effector population and either age or sex. Peripheral blood samples from the subjects in this study were obtained after obtaining written informed consent. The study was reviewed and approved by the University of Miami Institutional review Board (protocol 1995-0119).

In our preliminary work, the lymphocytes of 26 control, 12 long-standing T1D, and 7 recent T1D onset (<2 years) subjects were analyzed for NK cell frequency and subpopulation type. Total NK cells and NK effectors were compared among the three groups using non-parametric Kruskal–Wallis analysis followed by Dunns *post hoc* testing. Our preliminary data in T1D patients with long-standing disease provide evidence for a significant defect of both bulk NKs and the same NK effector phenotype (CD3^−^, CD14^−^, CD19^−^, CD66b^−^, CD7^+^, CD56^dim^, CD16^+^, CD27^−^, CD11b^+^; expressed as % of total lymphocytes). Moreover, we find a similar defect in at-risk autoantibody positive subjects, suggesting diminished NK effector populations and activity before diabetes diagnosis that may be an important component of the disease pathogenesis (Figure 3A). Of note, this observation holds when long-standing T1D patients were compared to age/sex-matched control subjects using the non-parametric Mann–Whitney *U* test. (Figure 3B; *P* = 0.0026). Importantly, no significant correlation was found between subject age or sex and NK status.

Natural killer dysfunction in the literature is shown to lead to chronic, subclinical infection from many viruses that have high prevalence in the general population (46, 56, 79, 80). This is the result of an inefficient clearance that is further exacerbated by the ability of these viruses to manipulate NK cells. This combination of critical defects might lead to the hyper-inflammatory adaptive cell response observed in patients, which in those with HLA variants predisposing to T1D could lead to the triggering of islet autoimmune responses and the chronic destruction of pancreatic β-cells (81). Given the major role of NK cells in the innate immune system and their interplay with the adaptive system, modulating the activity and function of downstream role players, such as NK-T cells, CD8^+^ cytotoxic lymphocytes, and T-regs, it is not an unreasonable proposition that NK cells may have a much bigger, upstream function in the etiology of T1D and many other autoimmune pathologies (13, 81). Recently, it has been demonstrated that NK cells have memory capabilities and with secondary exposures to pathogens increased IFN-γ secretion (82, 83). This suggests that the aberrant IFN-γ secretion observed in T1D and the hyper-inflammatory adaptive response drive by CD8^+^ CTLs could be the result of a cyclical response to lytic and lysogenic viral phases. This would correlate with the relapsing and remitting cycles characteristic of multiple sclerosis as well. Defective NK function and receptor activation is critical to maintenance of innate/adaptive balance and proper immune function, as evidenced in a recent study by Cook et al. where NK dysregulation lead to amplified aberrant responses, cytokine storm, and death (81). This further supports the potential greater role for NK cells in T1D development.

## Folic Acid, NK Viral Response, and T1D: Tying it all Together

Normal response to a primary viral infection occurs in three distinct phases. The first is an early, non-specific response characterized by fever, inflammation, and the production of interferons (Type 1: alpha, beta, epsilon, kappa, and omega, produced by fibroblasts and monocytes; Type 2: gamma, NK cells, and Th cells). There is a third interferon type with a role in specific types of infections, but primarily 1 and 2 are critical in both regulating, signaling, and activating the viral response. NK cells play a major role in this early response actively lysing cells recognized as non-self through the production/secretion of granules containing granzyme B and perforin. In addition, NK cells are intertwined with the activation and regulation of dendritic cell (DCs) activity in a positive feedback loop. They can directly activate DCs, dependent on TNF alpha and IFN gamma secretion. In turn, activated DCs can then further stimulate NK activity by secreting IL-12, IL-15, and IL-18. NK cells also regulate DC antigen presentation by actively lysing immature DC cells while sparing mature/active DCs. NK effectors then work to directly lyse virally infected cells while DCs circulate to stimulate adaptive responses through either the T-cell receptor mediated MHC Class II antigen presentation pathway (CD4^+^ Th cells) or MHC Class I antigen presentation pathway (CD8^+^ CTL cells). In addition to controlling DCs and antigen presentation, the NK cells of the regulatory phenotype (CD56^bright^, CD117^+^) modulate CD8^+^ CTL activity to balance responses through acquisition of a lytic phenotype and destruction of the CD8^+^ CTLs. In the aforementioned work of Dotta et al., where NK cells were observed in T1D post-mortem pancreatic sections of islets with no evidence of T-cell infiltrate but the presence of VP1, this might be explained by viral manipulation of innate responses. Recruitment of these CD56^bright^, CD117^+^ cells of a regulatory phenotype with lytic capacity would prevent CD8^+^ CTLs from tissue destruction through direct lysis of the infiltrating cells. This is further supported by the observation of islets containing CD8^+^ CTL infiltrates with no VP1 and no NK cells. A defect in NK number and activity, particularly in this CD56^bright^, CD117^+^ population would also adversely affect antigen presentation as immature DCs would not be targeted as effectively. This could lead to improper or excessive presentation, a hallmark of T1D, and amplified CD8^+^ CTL responses.

In our preliminary data (Figures 3A,B), we observed a drop in bulk NKs in long-standing and at-risk T1D subjects relative to controls and in the effector population responsible for viral clearance. This could have a twofold consequence. The decreased number of NK effector cells would lead to persistent viral infections and improper innate response. Over time, we hypothesize that the adaptive response is still activated through constant cycling of viral activation and latency. Adaptive CD8^+^ CTLs are activated and respond to the sites of persistent infection. Given the drop in bulk NKs, we theorize there is also a shortage of the CD56^bright^ regulatory cells that function to keep balance in this adaptive response. This likely leads to the infiltration and destruction of beta cells. The temporal progression to T1D onset is highly variable likely due to age of exposure to or reactivation of viruses, innate immune status at time of infection/reactivations, and exposure to environmental factors, in this case our proposed, folic acid. The folate pathway is instrumental in the production of molecules that fuel the activation and suppression of the immune response. Potential dysfunction in several segments of folic acid metabolism, detailed in the next paragraphs, could have direct impact on proper immune function and lead to T1D and T2D, if uncorrected.

Dysfunction in DHFR (Figures 1A,B) could result in dysfunction in the purinosome enzymes, resulting in increased intracellular adenosine and in turn an immunosupressive effect similar to that imparted by Methotrexate. Furthermore, this would cause a decreased level of THF, the substrate for both sides of the folate cycle, through the formation of 5,10 methyltetrahydrofolate (5,10-CH2 = THF); this would also result in defects in the homocysteine to methionine reaction and the associated enzymes. In critical support of this contention, metabolomic studies showed that plasma methionine was significantly lower in children at-risk for T1D compared to age-matched controls (84). A potential defect in the folate pathway is one explanation for this. In similar metabolomic studies examining differences between diabetic and non-diabetic NOD mice, pathway analysis indicated a deficiency of methionine in diseased animals, coupled with significant differences in several NK cell pathways, apoptosis pathways, purine and pyrimidine pathways and the DNA replication pathway, all important components of the folate pathway. This lack of THF would likely lead to increase in Betaine S-homocysteine methyltransferase (BHMT) activity, a redundant enzyme in the homocysteine to methionine reaction and a concomitant decrease in methionine synthase (MTR, MTRR) activity (Figure 1B, red box). A study that examined hypomethylation in diabetic rats relative to age/sex-matched controls reported significantly higher levels of BHMT activity and significantly lower methionine synthase activity, further suggesting folate pathway defects in diabetes (85).

When DHFR activity is suboptimal, it is possible that THF, normally utilized by the two major components (Figures 1A,B) of the folate pathway, is not produced properly. This would result in aberrant increased purinosome activity (PPAT, GART, and ATIC) and dysfunction in ADA, as in immunosuppression with Methotrexate, as shown in a 2006 paper (86). Increased activity within this complex has several effects, including dyslipidemia, internalization of the insulin receptor, and suppression of ADA (6). In normal metabolism, these are necessary biofeedback sensors. In a dysfunctional state, this results in an accumulation of intracellular ATP and adenosine, both adverse to proper cell function. Intriguingly, insulin receptor internalization is an established characteristic of both Type 1 and Type 2 diabetes. An excess of adenosine and a significantly lower level of ADA in lymphocytes, present with Methotrexate use, is a potent immune-suppressor of NK cell cytotoxicity and all immune cell function.

The production of inosine is stopped through the suppression of ADA, which also decreases NK cytotoxicity and proliferation. Intracellular ATP also increases. Studies have shown that high levels of intracellular ATP are a biofeedback signal driving apoptotic pathways (87, 88). This, would in turn, result in elevated serum extracellular ATP (eATP) released from cells undergoing apoptosis and through signaling pathways. eATP, at sufficiently high concentrations is a well-established signaling molecule that drives cascades of inflammation through cellular P2 receptors and is indicated in both acute and chronic/autoimmune inflammatory pathologies (89). Preliminary data from our own group show a significant increase in eATP at the onset of diabetes in NOD females relative to earlier time points in disease progression and to non-progressing NOD females. Increased intracellular ATP in NK cells leading to increased eATP could be one possible explanation for the observed deficit in NK number in T1D subjects relative to age- and sex-matched controls.

The upsurge in diabetes prevalence seen in Figure 2 includes patients diagnosed with Type 2 DM. Although associative, this increase could also be related to consumption of folic acid, given the multiple cellular functions mediated by the folate cycle. This is supported by a recent paper that demonstrated that excess B vitamin intake, the family containing folate (B9), was correlated with increased obesity and diabetes in the studied populations, although not attributed to any particular B vitamin (6). As folic acid is critical in development and its deficiency is correlated with increased incidence of neural tube defects during fetal development, it is one of the primary micronutrients in baby formula and prenatal vitamins. Folic acid and its derivatives are co-factors for the majority of cellular single-carbon reactions, including DNA methylation. Disruption of the folate cycle could, therefore, result in epigenetic changes from conception, onward.

**Figure 4:**
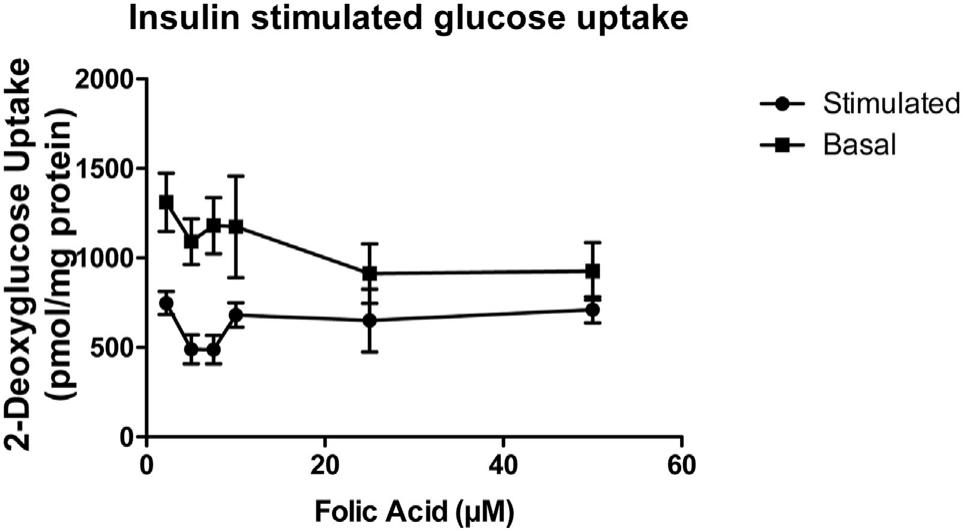
Insulin-stimulated glucose uptake of L6 myotubular cells exposed to sequential dosing of folic acid (*n* = 4). Insulin-stimulated glucose uptake was significantly different from basal uptake in all doses of folic acid (lowest dose 2.2 μM present in media) except 25 and 50 μM. Differential uptake decreased in a dose-dependent fashion.

Our preliminary data suggest that folic acid negatively affects glucose metabolism and confers a phenotype of insulin resistance both *in vitro*, in muscle cell lines and in animals supplemented with increased doses of folic acid. L6 rat myoblasts were cultured and differentiated into an insulin-responsive myotubular phenotype for 7 days (with and without increasing concentrations of folic acid) and utilized for insulin-mediated glucose uptake assays. The results are shown in Figure 4. With the exception of cells exposed to 25 and 50 µm folic acid, the cells displayed significant differences (*P* < 0.05) between basal and stimulated glucose uptake. Significance decreased in a dose-dependent fashion at concentrations greater than 7.5 µm FA (multiple paired *t*-test, *n* = 4 per group). This suggests that prolonged exposure of these muscle cells to elevated levels (>4× basal) of folic acid results in metabolic dysfunction indicative of an insulin resistant phenotype.

**Figure 5:**
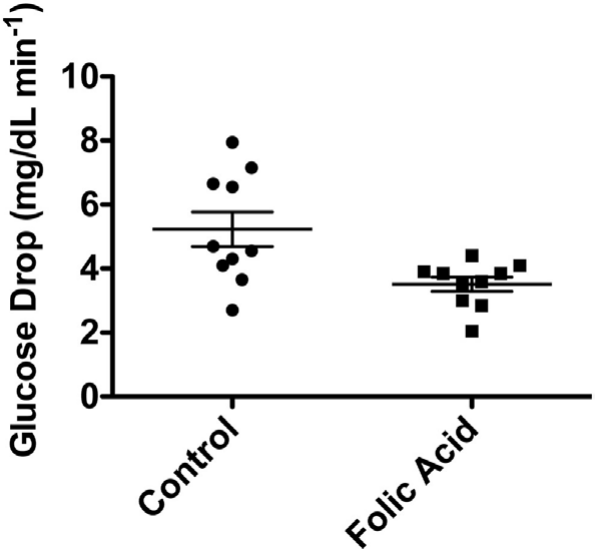
Decrease in blood glucose (mg/dL/min) in C57BL/6 mice after IP injection of insulin. Ten mice were supplemented with high-dose folic acid in their drinking water for 12 weeks prior to the insulin tolerance test. Blood glucose decrease was significantly slower in the group fed high-dose folic acid (*P* = 0.0125, non-parametric Mann–Whitney test).

Female C57BL/6 mice (*n* = 10 per group) were randomly assigned to two groups, control or HFA. HFA was solubilized in drinking water with dosing based on the average daily water consumption described in the literature (90). The RDA for mouse intake of folic acid per the American Institute of Nutrition is 2 mg/kg. The average water intake in C57BL/6 is 6.67 mL. The HFA group received 20× the standard dose, or 40 mg/kg, per other published studies (91). The supplementation with HFA began at 5–6 weeks of age and studies were performed at 20 weeks of age. Over the first 20 min after administration of insulin (3 U/kg body weight), the control group had a significantly faster decrease, shown in Figure 5, in plasma glucose levels relative to the group receiving the HFA treatment (5.23 ± 1.72 versus 3.51 ± 0.69 mg/dL/min; *P* = 0.0125, two-tailed non-parametric Mann–Whitney test). These data suggest an insulin-resistant phenotype related to high folic acid (HFA) intake.

The cause of NK cell dysfunction and its increased prevalence among the population not only of the United States but also worldwide remains undiscovered. All of the findings detailed above suggest an environmental factor that still eludes researchers despite many suggestions over the years ranging from heavy metals to chemical toxins and including viruses, more recently. These are likely secondary to a primary causal agent that adversely affects cellular metabolic pathways, protein synthesis, proliferation, and immune function. It is our hypothesis that many of these pathologies can be tied to the synthetic form of vitamin B9, folic acid, as all of the critical cellular functions listed above are directly modulated by the cellular folate pathway.

The folate cycle is critical in the maintenance of numerous cellular pathways and is an important site of cellular one-carbon metabolism/methylation. Given the importance of methylation in phenotypic expression through mechanisms of epigenetic modification, the folate cycle may have a role in the etiology of multiple pathologies. As it broadly affects all cells in the body, it can adversely impact multiple systems by slowing cellular metabolism reactions, accumulating unwanted reaction byproducts and disrupting homeostasis. As our hypothesis suggests, this could result in immune dysfunction leading to viral reactivation, improper antigen presentation and cytokine production, and unmodulated/unbalanced adaptive-heavy, CD8^+^ CTL and B-cell autoimmune responses in subjects with genetic predisposition. Therefore, folic acid could have a role in the development of T1D, along with many other autoimmune pathologies. The defects in insulin-mediated glucose metabolism that we have observed in our preliminary data suggest that folic acid could be a contributor to the recent upsurge of dyslipidemia, insulin resistance, obesity, and T2D. It is clear that there is strong need for further research into the folate cycle, its metabolites and the role these cellular pathways may have in the maintenance of immune function, metabolism, and general health status. Importantly, research into the innate system, critical in viral immune response, should be an area of greater focus as the evidence for a viral etiology is growing. The innate system evolutionarily precedes and is the upstream initiator of most adaptive responses including those that result in beta cell destruction in T1D. Improper innate function is likely the cause of downstream adaptive abnormalities that are the subject of the majority of immunological research in T1D.

If folic acid is indeed an environmental contributor to autoimmune and metabolic pathologies, as increasingly suggested, further research could tie this important cellular pathway to multiple disease etiologies and to conditions resulting from chronic innate immune deficiency such as cancer. It is encouraging to think that a simple dietary change may positively affect some of these conditions but the caveat is that anything that simply restores the innate effector function may result in strong NK-driven responses to viruses that lead to cytokine storm and further autoimmunity, if viruses are indeed a causal agent (81). This could be one possible explanation for the autoimmunity observed with cancer immunotherapies as enhanced responses to cancer could awaken innate responses once the immunosuppressive strategies of tumors are removed with cell destruction (92). Ideally, therapies to treat autoimmune conditions, particularly T1D, should not be immunosuppressive as this could lead to viral spread and the development of long-term pathologies, such as cancer. Rather, increased research into anti-viral strategies and gradual restoration of the innate balance to prevent catastrophic inflammatory responses (cytokine storm) might have better long-term outcome than current clinical trials.

## Ethics Statement

This study was carried out in accordance with the recommendations of HIPAA regulations from the University of Miami Leonard M Miller School of Medicine Institutional Review Board with written informed consent from all subjects. All subjects gave written informed consent in accordance with the Declaration of Helsinki. The protocol was approved by the University of Miami Leonard M Miller School of Medicine Institutional Review Board (protocol 1995-0119). For animal studies: this study was carried out in accordance with the recommendations of the Guidelines for the Care and Use of Laboratory Animals from the National Research Council Institute for Laboratory Animal Use and AAALAC. The protocol was approved by the University of Miami Institutional Animal Care and Use Committee.

## Author Contributions

CF and AB were responsible for the experimental design, data collection and analysis, literature review, writing, and editing of the manuscript.

## Conflict of Interest Statement

The authors declare that the research was conducted in the absence of any commercial or financial relationships that could be construed as a potential conflict of interest.
